# Nurses’ Knowledge, Attitudes, Confidence, and Practices with Genetics and Genomics: A Theory-Informed Integrative Review Protocol

**DOI:** 10.3390/jpm12091358

**Published:** 2022-08-24

**Authors:** Rebecca Puddester, April Pike, Joy Maddigan, Alison Farrell

**Affiliations:** 1 Faculty of Nursing, Memorial University of Newfoundland, St. John’s, NL A1B 3V6, Canada; 2 Health Sciences Library, Memorial University of Newfoundland, St. John’s, NL A1B 3V6, Canada

**Keywords:** genetics, genomics, nursing, change theories, health knowledge, attitudes, practice

## Abstract

Introduction: As key healthcare providers, nurses require genomic competency to fulfil their professional obligations in the genomic era. Prior research suggests that nurses have limited competency with genomics-informed practice. Concepts in the Rogers’ Diffusion of Innovation (DOI) theory (i.e., knowledge, attitudes, and attributes of innovation adopters) provide a framework to understand the process of adoption of innovations, such as genomics, across organizations. We aim to synthesize what is known about the adoption of genomics across nursing within the DOI framework to identify gaps and opportunities to enact sustained adoption of genomics in nursing. Methods and analysis: An integrative literature review, following Whittemore and Knafl’s five steps, will be conducted to evaluate qualitative, quantitative, and mixed-method primary studies that meet inclusion and exclusion criteria. The MEDLINE, PsychINFO, CINAHL, Cochrane, and Sociological Abstracts electronic databases will be searched in addition to the ancestry search method. Two researchers will perform independent screening of studies, quality appraisal using the Mixed-Methods Appraisal Tool, and data analysis using the narrative synthesis method. Disagreements will be resolved by a third reviewer. Findings in this review could be used to develop theory- and evidence-informed strategies to support the sustained adoption of genomics in nursing.

## 1. Introduction

The Human Genome Project (HGP) was a historic, international collaboration to map the entire human genome. Completed in 2003, the HGP was the origin of many subsequent genetic/genomic (GG) discoveries that improved understanding of human health and disease development [1,2,3]. While genetics is the study of individual genes, genomics is the study of an organism’s entire set of genes (genome) and the interaction of the genome with the environment [4]. Since the HGP, screening and prevention recommendations emerged that are proven to reduce cancer risk in individuals with inherited genomic predisposition to cancer [5,6]. GG discoveries have led to the development of precision medicine, where gene testing is used to predict drug responses based on an individual’s genetic/genomic makeup [7]. In precision oncology, therapeutics targeted at the genomic characteristics of cancerous tumours have resulted in increased survival and reduced treatment side effects [8,9]. With a projected 47% increase to 28.4 million cancer cases globally by the year 2040 [10], the clinical translation of GG advances could curtail the forecasted global cancer burden.

GGs play a role in the etiology of nearly every health condition; thus, the benefits of GG discoveries are revolutionary to healthcare. These benefits will only reach society through a GG-competent healthcare workforce [11,12]. Yet, the speed of GG discoveries is outpacing the speed at which healthcare providers are prepared to adopt these innovations into clinical practice [13]. Patients who accessed GG care have reported that their healthcare providers were insufficiently prepared to coordinate their long-term GG risk management [14]. Nurses comprise the largest group of healthcare providers globally and they play a central role in the coordination and continuity of patient care in the healthcare system. In general, outcomes of GG-informed nursing care are underexplored areas of research. However, nurses with GG competency have improved timely access to effective precision cancer treatment in cancer care pathways [15]. To meet patient expectations of their healthcare across the lifespan as we move forward into the genomic era of health, nurses must be equipped with GG competencies.

In 1962, less than a decade following the discovery of DNA, the inclusion of genetic content in nursing curricula was identified as a priority action for the profession [16]. There have been numerous calls in the literature since that time to accelerate the adoption of GG across nursing practice. Countries leading in genomic integration such as the United States (US) and the United Kingdom (UK) have made considerable progress in GG integration across nursing. In the UK, this was evidenced by the development of nursing GG competency statements [17,18], clearly defined roles for nurses in the new genomic medicine alliances unveiled across the UK [19], and nurse-led GG care pathways, such as the familial hypercholesterolemia service [20]. In the US, there are GG educational competencies and curricula guidelines for baccalaureate [21] and graduate level nurses [22], as well as nurse-led GG health policy recommendations [23,24]. Nurse leaders from these countries were also at the forefront of the creation of the Global Genomics Nursing Alliance (G2NA) [25] in 2017, an international collaborative network founded to promote GG literacy and integration across the global nursing community. 

While nurse leaders in the US, UK, and other countries have made significant contributions to advancing GG, there continues to be great variation in the global uptake of GG in nursing practice [26,27,28]. Ten years ago, in a mixed-methods systematic review of international literature, Skirton et al. [29] found that nurses’ competence in genetics was lacking; Wright et al. [30] conducted an updated integrative review and found little evidence that nurses’ competence in GG had improved in the five years following the initial review by Skirton et al. [29]. In a recent scoping review of GG educational interventions for nurses [31], it was found that while GG interventions were effective in improving nurses’ GG competency, further studies with improved methodological quality and evaluation of long-term outcomes are needed.

In the time since the preceding reviews [29,30], genomic science has continued to propel forward at a rapid pace while costs of gene sequencing continue to decrease [32]. Direct-to-consumer genetic/genomic testing is becoming increasingly popular [33], and there is growing public interest in whole genome sequencing [34]. In 2021, the UK unveiled its national genomic medicine alliance, where genome and multi-panel testing are now mainstreamed into routine healthcare across the entire country for many health conditions [35]. Stark et al. [36] described fourteen national government-funded genomic medicine initiatives to improve overall population health. We are nearing the cusp where GG are non-negotiable nursing competencies if nurses are to remain relevant, participate in interdisciplinary teams, and continue to meet their professional obligations to individuals, families, and communities. Thus, an up-to-date and comprehensive picture is needed of the state of adoption of GG across nursing, as well as an understanding of what factors are contributing to (or inhibiting) the current state of adoption of GG across nursing practice. This knowledge can be used to inform future priority research and strategies aimed at preparing nurses to be competent healthcare team members in the delivery of GG-informed healthcare.

### 1.1. Diffusion of Innovation Theory

Implementation frameworks can be useful to inform strategies to overcome gaps and delays in the translation of research discoveries, such as GG, into real-world practice [37]. Rogers’ [38] diffusion of innovation (DOI) is a theoretical framework that has been used for over sixty years to understand key concepts in the innovation adoption process across social systems or organizations. According to Rogers, an ‘innovation’ refers to a practice, idea, or object that is considered new to an individual or group. Rogers [38] defines ‘adoption’ as a five-stage process including (1) knowledge, (2) persuasion, (3) decision, (4) implementation and (5) confirmation. The DOI also outlines factors that contribute to the adoption of an innovation, such as variables in the social system, as well as the personal attributes of the adopter and their attitudes towards the innovation (i.e., concerning its relative advantage, compatibility, complexity, trialability, and observability). Rogers [38] noted that to proceed through the stages of implementation and confirmation, the adopters’ knowledge and attitudes surrounding the innovation are important factors in the decision-adoption process.

The DOI has been applied in previous studies examining the adoption of GG in nursing [39,40]. Calzone et al. [41] developed a psychometric instrument (i.e., the Genetics and Genomics Nursing Practice Survey [GGNPS]) designed to measure adoption of GG in nursing practice, and developed operational definitions for the concepts of attitudes, knowledge, confidence, and practices. These definitions were based on conceptual definitions in the DOI [38] (see Table 1). The DOI has also been used as a theoretical underpinning in other areas of nursing research, such as the identification of predictors associated with the adoption of evidenced-based practice in nursing students [42].

Another important concept in the DOI [38] is Rogers’ categories of innovation adopters which include: (1) innovators, (2) early adopters, (3) early majority, (4) late majority, and (5) laggards. According to Rogers, there are distinguishing characteristics of earlier and later innovation adopters; he described the ‘early adopters’ category as an important cohort when considering strategies to promote the widespread adoption of an innovation. As noted by Andrews et al. [39], the identification of early adopter characteristics offers “a means of identifying adopters and individuals who could then be targeted to influence and engage others who are less eager to change” (p. 880). Thus, definitions and assumptions in the DOI provide a foundation to synthesize what is known in the literature about the current state of adoption of GG, for the purpose of enacting sustained adoption of GG in nursing.

### 1.2. Aims

To our knowledge, there have been no reviews conducted since 2018 synthesizing nurses’ knowledge, attitudes, confidence, and practices surrounding GG. Given the speed at which genomics continues to advance, it is critical to assess if progress in nursing is aligned with these ongoing GG developments. This is especially timely, given the developments in the global nursing community since the time of the previous review [30], e.g., the establishment of the G2NA. Moreover, to our knowledge, no reviews surrounding nurses’ competence in GG have been specifically informed by the complex interplay of the concepts in the DOI theory. By considering (a) the current gaps in the diffusion of GG across the nursing profession, and (b) the characteristics of early adopters of GG in nursing, the identification of targeted strategies to promote the integration of GG in nursing will be strengthened. Although the focus of this review will be on nurses’ adoption of GG, consistent with Rogers’ theory, we acknowledge the influence of social systems (including organizational structures and interdisciplinary processes) as key factors in the adoption of GG among nurses. While examining nurses’ GG competency is a necessary first step, without accounting for the broader social context, the effectiveness of potential strategies to promote the application of GG in the health care system will be limited. Thus, a comprehensive and effective strategy to integrate GG into nursing will also require examination of the influence of other variables in the social system. If the findings of this review suggest that nurses have made minimal progress since 2018 in the adoption of GG, this will point to a need to examine further variables both internal and external to the nursing discipline and how they the influence the adoption of GG. The purpose of this review is to synthesize and analyze the scholarly literature on nurses’ knowledge, attitudes, confidence, and practices surrounding GG-informed care within the framework of the DOI, and to determine the characteristics of nurses who have adopted GG in their practice. However, if nurses in this review identify other factors in the wider social systems that are influencing the adoption of GG, these incidental findings may guide areas for future inquiry in research. Therefore, in this review, we will aim to answer the following review questions:

1. What are nurses’ (baccalaureate-prepared nurses, registered nurses, advanced practice nurses, nurse midwives, nurses in specialty areas of practice, nurse educators) attitudes, knowledge, confidence, and practices surrounding genetics and genomics?
2. What are the characteristics of nurses who have adopted GG in their practice?

## 2. Methods

To answer the research questions, the integrative review methodological framework of Whittemore and Knafl [44] will be used. This review methodology was selected as it is theoretically flexible and is a suitable approach to a literature review of primary studies when there is diversification in the phenomenon of interest; given the multiple concepts of the DOI [38] and the GGNPS [41] that were used to direct the approach to this review, the methodology [44] was deemed appropriate. The five-stage process of Whittemore and Knafl [44] will be followed: (1) problem identification, (2) literature search, (3) data evaluation, (4) data analysis, and (5) presentation. 

### 2.1. Problem Identification

As described in the introduction, efforts aimed at improving nurses’ GG-informed competency are not new endeavors in the profession. However, there is ongoing evidence of GG competency deficits among nurses [29,30]. Publications from the G2NA suggest that nurses in most countries are still in pre-contemplation or awareness and planning stages of GG integration [45]. Moreover, even in countries such as the US, with mature GG infrastructure and leadership initiatives, dense curricula and lack of GG competence among nursing faculty have been identified as persistent barriers to GG integration [46,47]. Therefore, we propose examining the current state of adoption of GG within the concepts of an established theoretical framework. For our first research question, we drew on both the conceptual domain definitions by Rogers [38] and the conceptual and operational domain definitions by Calzone et al. [41] concerning nurses’ (1) knowledge, (2) attitudes, (3) confidence, and (4) adoption (practices) in GG (see Table 1). As these conceptual and operational definitions are based on the DOI theoretical model, they provide a useful framework for the extraction of data and synthesis of knowledge in this review for the purpose of enacting change. Our second research question was also guided by the DOI [38] concepts concerning categories of adopters (i.e., early adopters and laggards). We will include studies published from 2003 onward (coinciding with the completion of the Human Genome Project). To avoid duplication of prior reviews, studies will be excluded if they were previously reported in the integrative and systematic reviews on the topic conducted in 2012 [29] and 2018 respectively [30]. The sample, phenomenon of interest, design, evaluation, and research type (SPIDER) framework [48] was used to develop the full list of inclusion and exclusion criteria (see Table 2).

### 2.2. Literature Search

Following the recommendations of Whittemore and Knafl [44], a minimum of two search strategies will be used: (1) a comprehensive electronic search, and (2) the ancestry approach [49]. To develop the strategy for the comprehensive electronic search, team members (RP, AP, JM) met with an experienced health sciences librarian (AF) to discuss the research question and purpose, and to select the appropriate electronic databases and search terms to be used in the integrative review. The search will be conducted in the MEDLINE, EMBASE, CINAHL, PsycINFO, Cochrane, and Sociological Abstracts electronic databases. AF conducted an initial search in MEDLINE on 1 June 2022, using search terms in combination with MeSH terms and Boolean operators (see Table 3). The search strategy was reviewed for suitability by the entire review team and peer-reviewed by a second health sciences librarian. MeSH terms ‘nurses’ and ‘nursing’ that explode to include all nurses in our inclusion criteria (e.g., nurse practitioners, nurse specialists) will be used. Furthermore, the use of the search term ‘nurs*’ with truncation is sufficiently sensitive to find all types of nurses in our inclusion criteria. Results from the search in each database will be uploaded into Endnote for duplication and then into Covidence by AF. As the second search strategy, the ancestry approach [49] will be used, where the reference lists of the eligible studies will be manually screened for primary studies that meet the inclusion criteria.

Two reviewers (RP, JM) will independently screen the title and abstract of all studies against the inclusion and exclusion criteria. Any conflicts at this stage will be resolved by discussion among RP and JM and, if necessary, a third reviewer (AP) will be involved until consensus is reached. Publications that meet inclusion criteria at the stage of title and abstract screening will go on to independent full-text review by the same two reviewers by comparing the studies against the inclusion/exclusion criteria. Again, any conflicts at the time of full-text review will be resolved through discussion between the two reviewers and if necessary, the third reviewer will be asked to provide input as a strategy to enhance rigor and minimize potential bias [49]. Studies that meet inclusion criteria at the time of full-text review will go on to the stage of data extraction.

A Preferred Reporting Items for Systematic Reviews and Meta-Analyses (PRISMA) flowchart [50] will be developed to provide a visualization of the study selection process. 

### 2.3. Data Evaluation

All articles will be critically appraised independently for methodological quality by two members of the review team (RP, JM) using the mixed-methods appraisal tool (MMAT) [51]. The MMAT is a valid and reliable tool for the appraisal of qualitative, quantitative, and mixed-methods studies, all three of which were included for evaluation in this review. Early in the appraisal process, the two reviewers will discuss and compare appraisal results and discuss any conflicts that arise, involving a third independent reviewer as necessary until consensus is achieved. For each study design in the MMAT, there are five appraisal questions with “yes”, “no”, and “can’t tell” as possible answers. For every appraisal criterion of “yes”, a numeric score of (1) will be assigned. Studies will be appraised as low quality when there are 2 or fewer “yes” answers out of a possible 5; appraised as medium quality when there are 3 “yes” answers out of 5; and appraised high quality when there are 4 or 5 “yes” answers out of a possible 5. For mixed-methods studies, we will appraise the overall study quality based on its lowest appraisal score of either the qualitative or the quantitative questions. Irrespective of the results of the methodological evaluation, all appraised studies will be presented in the integrative review both in-text and in summary tables and their overall quality rating will be reported in the summary tables.

### 2.4. Data Analysis

We intend to follow the five-stage synthesis process of Miles and Huberman [52] (as endorsed in Whittemore and Knafl [44]). This includes: (1) data reduction, (2) data display, (3) data comparison, (4) conclusion drawing, and (5) verification. The first reviewer (RP) will independently extract pertinent data and compile all data into literature summary tables. Information to be extracted in the tables include author, year, study design, study sample, intervention, key findings, study limitations and MMAT appraisal score. For qualitative studies, we will extract key participant quotes as part of the key findings. We will follow the approach as described by Aronsson et al. [53] that the second reviewer (JM) will extract data from 10% of the included studies into the summary tables and both reviewers will discuss and compare results as a strategy to enhance rigor. When all study tables are completed, the two reviewers will independently review the summary tables and compare the data for patterns, themes, and variations. To ensure rigor in this process, we will keep a documented audit trail of our thoughts and ideas. To minimize risk of bias, the two reviewers will come together to discuss themes and patterns and arrive at consensus of higher-level conclusions, involving the third reviewer as necessary. This approach will allow multiple, diverse perspectives on the data during the research process. In our analysis, we will ensure that as we are synthesizing higher level themes that all relevant concepts in the DOI theory that guided the research question have been addressed, i.e., nurses’ knowledge, attitudes, confidence, and practices surrounding GG, and the adopter [38] categories.

### 2.5. Data Presentation

As we expect heterogeneous results given the broad dimensions of the research questions, narrative summaries of the review findings will be reported. Data will also be summarized succinctly with summary tables (simplified versions of the summary tables developed in the stage of data analysis) in a peer-reviewed article.

Patient and public involvement: There was no involvement of patients or the public in the conceptualization or preparation of this review protocol.

Ethics and dissemination: Ethical approval is not required as only secondary data will be analyzed in this review. Findings will be reported in a peer-reviewed journal and presented at local, national, and international conferences to relevant audiences.

## 3. Discussion

Considering global trends towards genomics as the way of the future in healthcare [36], it is urgent to prepare nurses to provide competent GG-informed healthcare. There are growing numbers of evidence-based guidelines for the application of genomics in healthcare settings to improve outcomes and prevent disease [54]. The integration of these evidenced-based GG applications in routine care has the potential to significantly improve outcomes in morbidity and mortality for millions of people worldwide [55]. In this genomic era of health, patients will expect their healthcare providers to help them decipher the complexities of GG as they make decisions about their healthcare and will be disadvantaged if healthcare providers, including nurses, are unable to do so. This need to improve the GG competency of the nursing profession has been highlighted as far back as sixty years [16], and ongoing progress is needed to achieve a fully GG-competent nursing workforce, especially vis-à-vis the current speed of GG advances. A concerted effort is needed to consider the current state of adoption of GG in nursing within an implementation framework. This review will help inform effective strategies to promote and sustain a GG-competent nursing workforce.

### Strengths and Limitations of the Review

Possible limitations of this study include that the search strategy may not identify all studies examining concepts relevant to the focus of this review. Another limitation of this review that should be noted is that there may be bias in the selection and interpretation of studies. However, bias will be minimized through independent screening of the reviewers, discussion through consensus and involvement of a third reviewer as necessary to ensure multiplicity of perspectives. The use of English-language publications only is a limitation of this review; however, this was weighted against evidence from prior studies that exclusion of non-English studies has limited effect on overall evidence conclusions in literature reviews [56].

A strength of this review is that we will involve an experienced health sciences librarian to conduct the comprehensive search strategy. Three members of the review team (JM, AP, AF) have prior experience conducting comprehensive, systematic reviews. While previous literature reviews have been conducted on the topic, our comprehensive search strategy is likely to yield studies that may not have been retrieved in the prior reviews. Furthermore, in this review, we will provide an updated summary of studies published on the topic since 2018. Our review will be informed by a theoretical framework examining the adoption of innovations; therefore, knowledge translation is built into the design of our integrative review.

Other strengths to our design include the heterogeneity in study designs included in the review (qualitative, quantitative, mixed methods), allowing for the synthesis of rich data that captures multiple dimensions of the phenomenon of interest. Data will be evaluated with an appraisal tool (MMAT) known to be reliable and valid. The development of a stringent integrative review protocol will ensure transparency and reliability of the review process.

## Figures and Tables

**Table 1:** Conceptual and operational definitions of the DOI [38] and GGNPS [41].

DOI Domain	Conceptual Definitions (Rogers [38])	Conceptual Definitions (Calzone [41] p. 431, as Interpreting Rogers [38])	GGNPS Operational Domain Definitions (Calzone [41])
Attitudes	“Relatively enduring organization of an individual’s beliefs about an object that predisposes his or her actions… (frequently intervene between stages of knowledge and adoption)”. (pp. 174–175)	“Relative advantage offered by the innovation. Perceived attributes of the innovation, as well as contextual factors, such as identification of communication channels that facilitate or hamper adoption and sustained implementation”. (p. 431)	“Perceived importance, advantages, and disadvantages of integrating genomics into practice”. (p. 431)
Confidence		“Level of certainty that knowledge about the innovation is accurate *”. (p. 431)	“Confidence in discussing genetics with patients; deciding what family history information is relevant to assessing genetic susceptibility; availability, risks, benefits, and limitations of genetic testing; facilitating referral for genetic services”. (p. 431)
Competency/ Knowledge	“The individual or other decision-making unit learns of the innovation’s existence and gains some understanding of how it functions”. (p. 20)	“Recognition of the innovation and evidence of understanding the innovation and its function”. (p.431)	“Knowledge of the genomics of common disease, and the family history information needed to evaluate patients’ genetic susceptibility”. (p. 431)
Decision/Adoption (Practices)	“Decision to make full use of an innovation as the best course of action available”. (p. 473)	“Observation of use of the innovation”. (p. 431)	“Utilization of family history in the past three months **”. (p. 431)

* ‘Confidence’ is not defined in the DOI [38], however, Calzone et al. [41] developed a definition of confidence based on interpretation of Rogers’ description of personality traits that affect likelihood of adoption [43]; ** We will define ‘adoption’/practices beyond the Calzone et al. [41] definition. We will inductively determine the characteristics of practice adoption through our review.

**Table 2:** SPIDER framework domains for study inclusion and exclusion criteria.

SPIDER Categories	Inclusion Criteria	Exclusion Criteria
Sample	Studies in English. From any country. From 2003 onward. Focusing on nurses (including baccalaureate-prepared nurses, registered nurses, nurse educators, nurse midwives, advanced practice nurses, specialty practice nurses). Studies can be included if these target participants/respondents are at least an independent subgroup of a larger study and if this is identified. Studies prior to 2018 that were not reported in Skirton et al. [29] or Wright et al. [30].	Studies focusing on: Nursing students. Midwives (without specification of ‘nurse midwives’). ‘Healthcare providers’ with nurses included in that group, without distinguishing nurses as a subgroup in reporting/analysis.
Phenomenon of Interest	Studies focusing on conceptual definitions of the DOI/GGNPS as they relate to nursing and GG (examine at least one of the conceptual domains of interest: knowledge, confidence, attitudes, practices).	Studies focusing on the impact/effect of education interventions in nursing and GG on the conceptual domains.
Design	Primary research studies using any design.	Secondary research, theoretical papers, conference papers, discussion and opinion papers, grey literature.
Evaluation	Studies that examine/measure the GGNPS operational domains of interest as they relate to nursing and GG (studies do not have to specifically include or reference the DOI but must examine the concepts of interest). Qualitative studies, descriptive, quantitative studies (studies that measure at least one of the operational domains of interest).	Studies that evaluate the impact of educational interventions on DOI/GGNPS domains (knowledge, attitudes, confidence, practice).
Research Type	Qualitative, quantitative, or mixed-method primary studies.	

**Table 3:** MEDLINE draft search strings, 1 June 2022.

Search	MEDLINE Search Terms	Results
1	exp Genetics/	300,292
2	(genetics or genetic or hereditary or genomic or genomics).tw,kf.	1,358,549
3	genetics.fx.	3,813,389
4	1 or 2 or 3	4,398,091
5	exp Nurses/	95,486
6	exp Nursing/	260,834
7	nurs*.tw,kf.	506,930
8	5 or 6 or 7	655,510
9	4 AND 8	8334
10	exp “Attitude of Health Personnel”/or Attitude/	218,215
11	exp Health Knowledge, Attitudes, Practice/	123,603
12	exp Professional Competence/	126,495
13	Self Concept/	59,916
14	exp Self-Assessment/	13,161
15	Decision Making/	102,541
16	exp Clinical DecisionMaking/	14,421
17	exp Decision Making, Shared/	1593
18	(attitude* or confiden* or competen* or knowledg* or skill* or understand* or percept* or “family history” or “family tree” or decision* or practice*).tw,kf.	4,326,000
19	10 or 11 or 12 or 13 or 14 or 15 or 16 or 17 or 18	4,567,066
20	9 and 19	3389
21	limit 20 to yr = “2003–Current”	2692

## Data Availability

No new data were created or analyzed in this study.
